# New Insights in the Design of Bioactive Peptides and Chelating Agents for Imaging and Therapy in Oncology

**DOI:** 10.3390/molecules22081282

**Published:** 2017-08-02

**Authors:** Anna Lucia Tornesello, Luigi Buonaguro, Maria Lina Tornesello, Franco Maria Buonaguro

**Affiliations:** Molecular Biology and Viral Oncology Unit, Istituto Nazionale Tumori, IRCCS, Fondazione Pascale, 80131 Napoli, Italy; l.buonaguro@istitutotumori.na.it (L.B.); ml.tornesello@istitutotumori.na.it (M.L.T.); fm.buonaguro@istitutotumori.na.it (F.M.B.)

**Keywords:** peptide, chemical modification, peptide cyclization, d-amino acids, glycosylation, PEGylation, chelators, TETA, AAZTA, DOTA, TRAP, NOPO

## Abstract

Many synthetic peptides have been developed for diagnosis and therapy of human cancers based on their ability to target specific receptors on cancer cell surface or to penetrate the cell membrane. Chemical modifications of amino acid chains have significantly improved the biological activity, the stability and efficacy of peptide analogues currently employed as anticancer drugs or as molecular imaging tracers. The stability of somatostatin, integrins and bombesin analogues in the human body have been significantly increased by cyclization and/or insertion of non-natural amino acids in the peptide sequences. Moreover, the overall pharmacokinetic properties of such analogues and others (including cholecystokinin, vasoactive intestinal peptide and neurotensin analogues) have been improved by PEGylation and glycosylation. Furthermore, conjugation of those peptide analogues to new linkers and bifunctional chelators (such as AAZTA, TETA, TRAP, NOPO etc.), produced radiolabeled moieties with increased half life and higher binding affinity to the cognate receptors. This review describes the most important and recent chemical modifications introduced in the amino acid sequences as well as linkers and new bifunctional chelators which have significantly improved the specificity and sensitivity of peptides used in oncologic diagnosis and therapy.

## 1. Introduction

Natural or synthetic peptides are short chains of amino acids useful to analyze functions of the full length proteins and in particular the specific binding to cognate cell-membrane associated receptors and their subsequent internalization. The increased expression of peptide receptors in many human tumors allowed to develop a wide range of moieties useful for diagnostic imaging, cancer radiotherapy and immunotherapy [1,2,3].

**Figure 1:**
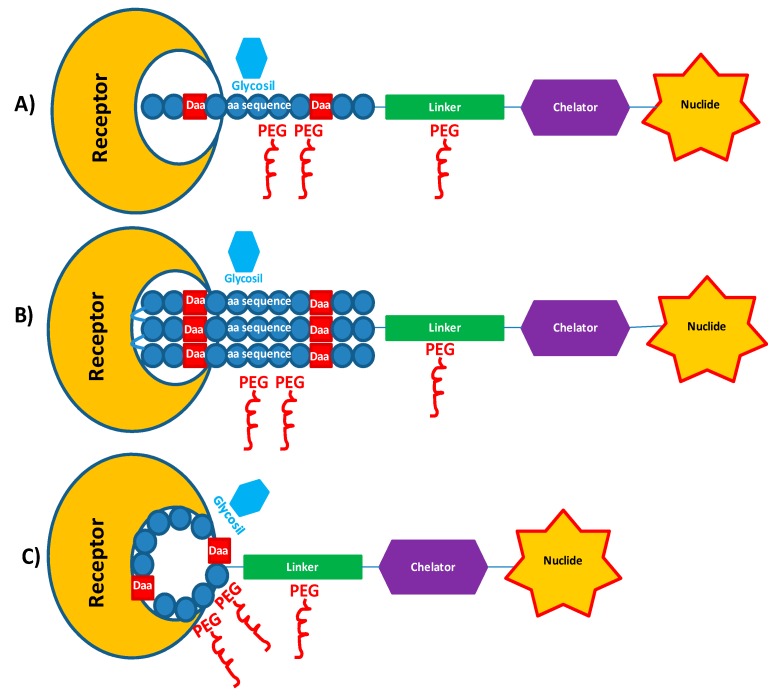
Schematic view of main chemical modifications (d-amino acids, Glycosylation, PEGylation, etc.) introduced in peptide-based radiopharmaceuticals designed for imaging or radiotherapy. The modified peptide moiety, covalently bounded to the chelator through a linker inserted as spacer, acts as carrier to specific receptor. (**A**) Linear targeting peptide; (**B**) Multimeric targeting peptide; (**C**) Cyclic peptide.

Since natural peptides have a short half life, due to their rapid degradation caused by about 600 different proteases in the human body [4], several strategies have been used to produce metabolically stabilized analogues while preserving the biological activities of the original molecules (Figure 1). Main chemical modifications include the synthesis of pseudo-peptides containing d-amino acids or more stable non-natural amino acids, modifications at the C- or N-terminus, multimerization, cyclization, PEGylation, glycosylation, etc. [5,6]. In Table 1 are listed the most relevant peptides used in medicine and their modified analogues.

Additionally, different chelators have been designed, synthesized and conjugated to synthetic peptides to improve the stability of radiolabeled molecules and their biodistribution in the human body [7].

In this review, we summarize recent advances in chemical modifications of amino acid sequences, linkers and chelators to produce optimal moieties for diagnosis and therapy of human neoplastic diseases.

## 2. Chemical Modifications of Synthetic Peptides

Optimization of peptide based drugs relies on their ability to bind specific receptors with high affinity, to permeate across biological barriers such as the intestinal lumen and mucosa as well as the blood-brain barrier, and to resist in vivo degradation [8]. The pharmacokinetics of natural amino acid sequences can be optimized through the introduction of (1) conformational constraints (i.e., induced by cyclization, or insertion of non-natural amino acids in the peptide sequences) providing unfavorable changes in the binding entropy; and (2) conjugation with glycosylated moieties or polyether compounds at the N-terminus end of synthetic peptides. Further strategies employed to increase the stability of peptides include the formation of dimers, tetramers or heterodimers which improve the stability and the affinity of synthetic peptide chains to their receptors [9,10].

### 2.1. Peptide Cyclization and Insertion of Non-Natural Amino Acids

Cyclic peptide structures are mainly due to the formation of disulfide bonds between the thiol groups of two unprotected cysteines within the linear peptide. Several cyclized peptides are currently used in nuclear medicine, such as somatostatin, RGD tripeptide, cholecystokinin and minigastrin, as well as bombesin and vasoactive intestinal peptide (VIP).

The somatostatin is a 14-amino acid peptide which binds to the five somatostatin receptor subtypes (sst 1–5) and regulates the release of several hormones [11]. A stable cyclic somatostatin analogue was obtained by introducing a disulfide bridge between Cys3 and Cys14 [AGCKNFFWKTFTSC (Cys-Cys cyclization)] while preserving the residues 7–11 essential for binding to receptors [12,13].

Moreover, an enhanced biological activity and reduced metabolic degradation was achieved by the synthesis of the octapeptide octreotide in which the insertion of the d-amino acid d-Phe at N-terminus and the amino-alcohol Thr-ol at C-terminus conferred a half-life of about 2 h in the human body [14]. The TATE and Y3-TATE somatostatin analogues, containing a free carboxylic group at C-terminus and Tyr amino acid in place of Phe in position 3, are characterized by a significant higher affinity to sst2 receptor (Table 1). Moreover, the incorporation of stable amino acids, such as β-DAP (β-(l-1,2-diamino propionic acid) and homocysteine (Hcy) in depreotide and (2-naphthyl)-d-alanine in lanreotide as well as the amidation of their C-terminus improved the tumor uptake in comparison to octreotide [15]. In a recent study, Martín-Gago et al. showed that the introduction of l-3-(3′,5′-difluorophenyl)-alanine (Dfp) in substitution of Phe in six new somatostatin analogues produced an electron-poor aromatic ring in the network of aromatic interactions which conferred new chemical features to the synthetic peptides [16]. Specifically, replacement of each Phe residue at position 6, 7 or 11 with Dfp and introduction of d-Trp at position 8 increased the peptide yield. The Dfp at position 7 resulted in a remarkable increased binding affinity to sst2 and sst3 receptors, while the synthetic peptides with Dfp at positions 6 or 11, particularly [d-Trp8,l-Dfp11]-SRIF (Pep 3 in Table 1), showed a selective binding to sst2 receptor, equivalent to the native somatostatin activity, and higher affinity than octreotide. On the other hand, the presence of the Dfp residue at position 7 (Pep2) conferred a higher affinity and selectivity for sst3 receptor. Such results demonstrated that Dfp amino acids are efficient building blocks in the synthesis of peptide analogues and open new possibilities for designing molecules with tuned selectivity and specificity towards sst2 and sst3 receptors [16].

The somatostatin analogs currently employed in nuclear medicine act as agonists of the native peptide and cause internalization of the receptor-ligand complex, which has been considered of high importance for optimal tumor uptake of radiometals. Recent studies have shown, however, that receptor antagonists, which are not internalized in the cells, are even better than agonists for tumor imaging [17,18]. The design of peptide antagonists requires several chemical modifications such as deletions or the introduction of non-natural amino acids with different chirality. In the octreotide scaffold the inversion of chirality at positions 2 and 3 was shown to cause relevant structural modifications converting a somatostatin agonist into an antagonist [19]. Ginj et al. firstly compared the radiolabeled sst2 BASS antagonist with the radiolabeled Y3-TATE agonist in vivo (Table 1) [20]. The antagonist showed a lower receptor affinity than the agonist but its in vivo tumor retention was nearly twice that of the agonist. Thereafter, several new somatostatin antagonists have been developed by the introduction of d-4-aminocarbamoyl-Phenylalanine (d-Aph(Cbm)) in place of d-Trp in position 8, and 4-amino-l-hydroorotyl-phenylalanine (Aph(Hor)) in position 7 in place of Tyr to improve the receptor affinity [18,21]. Fani et al. demonstrated that complexation of peptide antagonists with specific radiometals or replacing the chelator may dramatically change the receptor affinity and the in vivo distribution of radiotracers. The Ga-DOTA analogs have a binding affinity on sst2 receptor which is up to 60 times lower than the Y-DOTA, Lu-DOTA, or In-DOTA compounds. However, substitution of DOTA with NODAGA chelator caused a massive increase in the binding affinity [22]. The sst2 antagonist ^125^I-JR11, compared to the agonist ^125^I-Tyr^3^-octreotide, has shown to label many more sst2 binding sites than the agonist in tumor cells as well as in adjacent sst2-expressing tissues [23]. Indeed, Reubi et al. evaluated quantitatively the binding activity in vitroof sst2 antagonist ^125^I-JR11 and sst2 agonist ^125^I-Tyr^3^-octreotide in a large series of non-neuroendocrine and neuroendocrine tumors, including those with high density of sst2 receptor, such as gastroenteropancreatic neuroendocrine tumor and pheochromocytomas, and those with little or no expression of sst2 receptor, such as renal cancer, breast cancer, prostate cancer, non-Hodgkin lymphoma (NHL), medullary thyroid cancer and colon cancer [23]. Importantly, this study showed that the antagonist ^125^I-JR11 is attractive for targeting many tumor types besides neuroendocrine cancers. Nicolas et al. compared in preclinical studies the sst2-antagonist ^177^Lu-DOTA-JR11 with the sst2-agonist ^177^Lu-DOTA-TATE and observed that ^177^Lu-DOTA-JR11 exhibited higher tumor uptake, longer tumor retention and improved tumor-to-kidney ratio. Moreover, the mass-escalation study indicated that this antagonist may further improve the safety window of peptide receptor radionuclide therapy by reducing normal tissue (i.e., liver and bone marrow) dose. Clinical studies are warranted to confirm the higher efficacy and lower toxicity of ^177^Lu-DOTA-JR11 [24].

The integrins family comprises numerous transmembrane receptors regulating cell adhesion and interaction with the extracellular matrix. The integrins α_v_β_3_ and α_v_β_5_, highly expressed on many solid tumors but rare on normal cells, are recognized by several proteins containing the tripeptidel-arginine-glycine-l-aspartic acid (RGD) [25,26,27,28,29]. Both linear and cyclic RGD peptides have been evaluated as radiotracers for tumor imaging by SPECT or PET [30]. However, linear RGD peptides showed in general low binding affinity (IC_50_ > 100 nmol/L), lack of specificity to α_v_β_3_, and instability in the bloodstream due to the high degradation rate caused by the high susceptibility to proteases of the aspartic acid residue. Kapp et al. performed a comparative study on a large number of integrin-targeting molecules including linear and cyclic peptides, peptide-mimetics as well as commonly used reference compounds by using a standardized competitive ELISA-based test [31,32]. The ELISA-like solid phase binding panel comprised several integrin subtypes such as α_v_β_3_ and α_v_β_5_ (both bind vitronectin), α_v_β6 and α_v_β8 (bind LAP), α5β1 (binds fibronectin) and αIIbβ3 (binds fibrinogen) and has been useful to measure the inhibition of integrins binding to immobilized natural extracellular matrix ligands [32]. They observed that all tested linear peptides (including RGD, RGDS, GRGD, GRGDS, GRGDSP, GRGDSPK GRGDNP and GRGDTP) were active on integrins α_v_β3, α_v_β5, and α5β1, and selective against α_v_β6, α_v_β8 and αIIbβ3, although they were subjected to enzymatic degradation. Cyclization and incorporation of D-amino acid residues increased the stability and affinity of all tested RGD peptides to the receptors by reducing structural flexibility [33,34]. Several cyclic RGD peptides have been developed such as the c(RGDfV) with a high affinity for α_v_β3 and total selectivity against αIIbβ3, the c(RGDxX) containing a d-Phe or d-Tyr or d-Trp at the position 4 which is essential for preservation of the α_v_β3-binding affinity, and the most active methylated cyclic peptides (RGDf(NMe)V), named cilengitide, characterized by a half-life of about four hours [35]. Kapp et al. showed that cilengitide has a remarkably low IC_50_-value for α5β1. Interestingly, in their study the affinity for α_v_β3 (0.61 nM) and α_v_β5 (8.4 nM) integrins was the highest obtained among all synthetic peptides developed and studied to date [32]. The uptake and internalization of RGD peptides has shown to be improved by a multimeric structure especially in cancer cells with low receptor density [36]. Particularly, homodimers, homo-multidimers and heterodimers of different peptides are conjugated with suitable linkers to form moieties targeting multi-receptors expressed on tumor cells. Multimerization of RGD analogs is achieved by the substitution of Val residues with Lys useful for conjugation of different moieties (chelators, drugs or probes) to its side chain [37].

More recently, the integrin αvβ6 c(FRGDLAFp(NMe)K) has been found highly upregulated in tumors such as pancreatic, basal cell, cervical, gastric, colorectal, and non-small cell lung cancers as well as oral squamous cell carcinomas (Table 1), [38]. Notni et al. synthesized mono-, di-, and trimeric conjugated of αvβ6 integrin via click chemistry by using the new chelator TRAP, with asymmetrical azide substitution pattern, and an additional polyethyleneglycol (PEG) linker [39]. They observed that multimers improved αvβ6 integrin affinity but did not exhibit superior tumor accumulation in PET scans and showed inferior pharmacokinetics compared to the respective monomers [39].

The integrin α5β1 has been recently shown to be involved in the spreading of metastatic cells, resistance to chemotherapy and ionizing radiation as well as tumor aggressiveness [40]. A highly active antagonist of α5β1, namely (2,2′-(7-(1-carboxy-4-((6-((3-(4-(((*S*)-1-carboxy-2-(2-(3-guanidinobenzamido)acetamido)ethyl) carbamoyl)-3,5-dimethylphenoxy)propyl)amino)-6-oxohexyl) amino)-4-oxobutyl)-1,4,7-triazonane-1,4-diyl)diacetic acid, FR366) has been developed, coupled to the chelator NODAGA, labeled with ^68^Ga and tested in vitro and in vivo models [41]. The results showed high affinity for integrin α5β1, specific uptake in tumor cells and good imaging in small animals by PET/CT [41].

Cholecystokinin (CCK) and gastrin constitute a family of homologous peptide hormones (DYMGWMDF-NH_2_) with binding affinity to the gastrin/CCK-B receptor, whereas the CCK-A receptor is bound only by sulfated CCK-peptides [42]. The CCK receptor is over expressed in various cancer cells, particularly in lung and pancreatic cancers [43]. The cyclic CCK analogues bound to the universal macrocyclic chelator 1,4,7,10-tetraazacyclododecane-1,4,7,10-tetraacetic acid (DOTA), such as DOTA-K(glucose)-GWNleDF (DOTA-glucose-CCK) and DOTA-Nle-cyclo(EWNleDFK-NH_2_) (DOTA-[Nle]-cCCK), have been synthesized, radiolabeled with ^177^Lu and evaluated for their affinity to the receptor by a competitive binding [44]. The ^177^Lu-DOTA-[Nle]-cCCK DOTA-[Nle]-cCCK showed a higher affinity than ^177^Lu-DOTA-glucose-CCK to the CCK receptor expressed in cell lines [29].

In addition, bifunctional CCK molecules have been obtained as mixed aggregates of two amphiphilic linear monomers (CCK1 and CCK2) containing each the same lipophilic C18 chains but different moieties on the hydrophilic heads. Particularly, CCK1 contained the peptidic fragment recognizing the cell receptors, while CCK2 was bound to the chelating agent diethylenetriaminepentaacetic acid (DTPA)-Glu able to coordinate ^111^In [45]. However, these radiolabeled aggregates injected in nude mice showed high retention in both A431 epidermoid carcinoma xenografted cells as well as in the liver and kidneys probably due to the presence of lypophilic chains [30].

The minigastrin based radioligands (MG, l-(E)5-AYGWMDF-NH_2_), such as [^111^In-DOTA]MG0 ([^111^In-DOTA-DGlu1]-MG), showed higher affinity for the CCK2 receptor and good stability [46]. However, their clinical use has been hampered by the unfavorable high retention in the kidneys [47]. Several chemical modifications have been introduced in the MG peptide chain, such as deletion of Glu residues at position 2–6 or substitutions of l-Glu with d-Glu, in order to decrease kidney uptake [48]. Kaluodi et al. analyzed twelve different MG chemical modified peptides and showed that the [^111^In]CP04 ([^111^In-DOTA-DGlu^1−6^]minigastrin), coinjected with phosphoramidon (PA), was the better radiopeptide candidate for clinical validation in medullary thyroid carcinoma patients due to the optimal tumor-to-kidney ratios in animal models [49].

Bombesin is a neuropeptide hormone composed of 14 amino acids (EQRLGNQWAVGHLM-NH_2_), which has high binding affinity to the G protein-coupled gastrin-releasing peptide receptor (GRPR/BB2) [50]. The GRPR/BB2 regulates the release of gastrointestinal hormones, smooth muscle contraction and epithelial cell proliferation [51]. The bombesin is up regulated in several tumors such as prostate carcinoma, small-cell lung cancer, breast and pancreatic cancers [52]. Several chemical modifications have been introduced in the synthetic bombesin to stabilize the structure, to increase the binding affinity and to potentiate agonist or antagonist properties. Particularly, the methionine at the C-terminus (Met-14), sensitive to oxidation, and Leu-13 have been substituted with norleucine (Nle) and cyclohexylalanine (Cha), respectively, in order to improve the overall stability and binding affinity [53]. Other amino acids sensitive to endopeptidases, such as His-12/Leu-13 and Gly-11/His-12, have been substituted with non-natural Cha or statin (Sta) and *N*-methylglycine, respectively, to increase the in vivo stability [38]. Finally, the introduction of d-Phe at the N-terminus and Sta-13 in place of Leu-13 conferred antagonistic properties superior to the agonistic features in terms of higher tumor targeting, retention, and tumor-to-tissue ratios [54].

Vasoactive intestinal peptide (VIP), consisting of 28 amino acids (HSDAVFTDNYTRLRKQMAVKKYLNSILN), acts as a neuroendocrine mediator with an important role in water and electrolyte secretion in the gut [55,56]. C-terminal region of the VIP-28 peptide binds to the VIPAC1 and VIPAC2 receptor subtypes [57,58,59]. VIP is quickly metabolized in liver and excreted from kidneys thus chemical modifications extending the biological half-life have been very important in order to meet the imaging requirement of the radiolabeled peptides. Cheng et al. synthesized a VIP analogue, the [R^8,15,21^, L^17^]-VIP, exhibiting high stability, receptor specificity and fast elimination of ^18^F-labeled [R^8,15,21^, L^17^]-VIP in preliminary studies conducted in mice [60]. Such properties were conferred by the introduction of Arg in place of Asp-8, Lys-15, Lys-21 and of Leu in place of Met-17 in VIP sequence which rendered the analogue suitable for ^18^F-labeling and resistant to the proteolytic degradation [61].

α-Melanocyte-stimulating hormone (α-MSH) is a peptide hormone (Ac-SYSMEHFRWGKPV) affecting morphology, tyrosinase activity and melanogenesis in human melanoma cells. It binds to the melanocortin 1 receptor (MC1R) which is over expressed in mice and human melanoma metastases [62]. Four synthetic peptides containing non-natural amino acid Nle and d-Phe at different positions (Ac-GGNle-CCEH(d-F)RWC-NH_2_, Ac-GGNle-CCEH(d-F)RWCRP-NH_2_, Ac-CCEH(d-F)RWC-NleGG-NH_2_, and Ac-CCEH(d-F)RWCRP-NleGG-NH_2_) have been designed and their binding affinity to melanocortin-1 (MC1) receptor has been evaluated in B16/F1 melanoma cells [63]. The C-terminal moiety Arg-Pro was very important for the efficient binding to MC1 receptor while the N-terminus-Gly-Gly-Nle-moiety was essential in lowering liver and kidney uptake. The study of biodistribution showed that ^99m^Tc-Ac-GGNle-CCEH(d-F)RWCRP-NH_2_ peptide has the best imaging performance for the high tumor uptake and fastest urinary clearance [48].

Neurotensin (NT) is a 13 amino acids peptide (pEYENKPRRPYIL) targeting three different receptors (NTR_1_, NTR_2_, NTR_3_) over expressed in different human cancers including Ewing Sarcomas, meningiomas, astrocytomas, medulloblastomas and pancreatic carcinomas [64]. Natural neurotensin is rapidly degraded in the blood by endogenous peptidases, thus several modified analogues have been developed for diagnostic imaging in cancer. Fanelli et al. synthesized a neurotensin analogue containing the TMS Ala (silylated amino acid (l)-(trimethylsilyl)alanine) residue at the position 13 which increased hydrophobicity of the region comprised between amino acids 8–13 [65]. Such modifications rendered the NT(8–13) peptide a potent receptor agonist with improved binding affinity to the receptor and optimal in vivo biological functions [65]. Recently, Mascarin et al. obtained a substantial increase (70-fold) in the stability of the peptide in human blood by replacing the Ile-12 with Tle-12 [Tle=C(Me)3] in the binding sequence of neurotensin NT(8–13) [66]. The overall results of these studies suggested that modifications in the peptide’s backbone, more than amino acid exchanges, are the preferred strategy to achieve enhanced metabolic stability and retained receptor affinity of NT-based radiotracers for tumor-targeting strategies.

### 2.2. PEGylation and Glycosylation of Synthetic Peptides

The conjugation of carbohydrate molecules such polyethylene glycol (PEG) or *O*- and *N*-glycosyl units to peptides can significantly improve their pharmacokinetic properties by increasing the hydrophilicity while reducing the sensitivity to proteolysis and by decreasing renal clearance and their hepatic accumulation [67].

Dapp et al. synthesized and radiolabeled a series of PEGylated Bombesin (7–14) analogues with ^99m^Tc(CO)(3) and observed that derivatization of (N(α)His)Ac-Bombesin (7–14)[Cha(13),Nle(14)] analogue with linear PEG molecules of various sizes, such as 5 kDa [PEG(5)], 10 kDa [PEG(10)] and 20 kDa [PEG(20)] did not affect the binding affinity of Bombesin analogues for BN(2)/GRP receptors (K(d) < 0.9 nM) [68]. Moreover, PEGylation improved the stability of Bombesin conjugates in vitro and in vivo while the in vitro binding kinetic was slower compared to non-PEGylated analogues. The best pharmacokinetics in vivo was obtained with Bombesin analogues conjugated with PEG(5) molecule which showed a faster blood clearance, the preferential renal excretion and higher tumor uptake compared to non-PEGylated analogue [68].

More recently, Kapoor et al. designed the GIRLRG peptide, that specifically targets the glucose regulated protein 78 (GRP78) expressed in several cancers, and conjugated it with PEG to increase the stability. NanoSPECT/CT imaging of nude mice bearing heterotopic cervical (HT3), esophageal (OE33), pancreatic (BXPC3), lung (A549) and glioma (D54) tumors revealed that ^111^In-PEG-GIRLRG specifically binds cervical, esophageal, pancreatic, lung and brain tumors opening new opportunities to use PEG-GIRLRG peptide for human diagnostic imaging [69].

Hausner et al. studied the effect of dual (N- and C-terminal) PEGylation of the integrin αvβ6-targeting ^18^F peptide and observed that the size and location of the PEG units significantly affected αvβ6 targeting and pharmacokinetic [70]. Particularly, the bi-terminally PEGylated displayed the more favorable combination of high αvβ6 affinity, selectivity, and pharmacokinetic profile compared to C-terminal PEGylated and the un-PEGylated 18F-FBA-A20FMDV2 [70]. The two PEG units seemed to act synergistically to confer optimal αvβ6 tumor uptake and retention [70].

Glycosilation and pegylation of modified cyclic RGD c(RGDfK) has also shown to improve the pharmacokinetics. In particular, the F-Galacto-c(RGDfK) and c(RGDfK)-Peg-MPA) (MPA, mercapto propionic acid) showed IC_50_ of 100nM and 8–15 nM, respectively [71].

Glycosylation of CCK analogue, obtained by glucose binding to the Lys side chain at N-terminal region of the synthetic peptide, contributesto decrease its lipophilicity and to improve sensitivity, specificity, and pharmacokinetics in CCKR-expressing tumors [44].

## 3. Spacers, Chelators and Radionuclides

The most sensitive and non-invasive molecular imaging techniques are the radionuclide-based positron emission tomography (PET) and single photon emission computed tomography (SPECT). The radiotracers are metal complexes composed of a targeting molecule, such as a peptide, a linker able to modify the pharmacokinetic, a bifunctional chelator and a metallic radionuclide. The coordination chemistry of the radiometal influences the geometry and stability of the radiometal chelate. The radiolabeled moieties employed in diagnosis must have an half-life sufficient to carry out the chemical synthesis and to concentrate in the target tissues or organs while must be easily cleared from non target organs. They are generally injected at very low concentrations (10^−6^ to 10^−8^ M) and do not cause significant pharmacological effects [5,72].

### 3.1. Spacers

The spacers are inert molecules used to increase the distance of peptides from chelators in order to prevent steric influence and loss of activity on the cell receptors upon functionalization. In fact, the molecular size, lipophilicity, and the flexibility of the functional moiety can influence the binding of the bioactive peptide to its target [73,74].

The length and composition of the spacer groups as well as the chemical properties of the radiolabeled moiety influences the binding affinity of the radiopharmaceutical to the receptor, the accumulation of radionuclides in tumor cells and the pharmacokinetic. Hoffman et al. analyzed a series of DOTA-X-Bombesin[7–14]NH_2_ conjugates containing X = 0, beta-Ala, 5-Ava, 8-Aoc, or 11-Aun and labeled with In(III)/^111^In by competitive binding assays on human prostate cancer cells with IC_50_ values less than 2.5 nmol/L for analogs with beta-Ala, 5-Ava, and 8-Aoc spacers. The biodistribution studies of ^111^In-DOTA-X-Bombesin[7–14]-NH_2_ conjugates performed in mice showed that the uptake of radioactivity in the pancreas increased with increasing length of hydrocarbon spacer while the radioactivity was efficiently cleared by renal/urinary excretion [75]. Thus, radiolabeled DOTA-X-Bombesin[7–14]-NH_2_ moieties containing hydrocarbon spacers with 5–8 carbons represent good candidates for diagnostic and therapeutic radiopharmaceuticals [76].

Antunes et al. analyzed different spacers, based on 8-amino-3,6-dioxaoctanoic acid (PEG2), 15-amino-4,7,10,13-tetraoxapentadecanoic acid (PEG4), *N*-acetyl glucosamine (GlcNAc), triglycine, beta-alanine, aspartic acid, and lysine, between the chelator DOTA and the somatostatin analogue NOC [77]. They observed that in general the spacers marginally influenced the binding affinities to the hsst2 and hsst5 receptor subtypes but they observed an almost complete loss of hsst3 affinity of the [^111^In-DOTA]-X-NOC peptides.

Mascarin et al. performed a comparative analysis, based on cell internalization experiments, receptor affinity, biodistribution and blood serum stability, of several radiolabeled Neurotensin analogues to identify the optimal derivatives of NT(8–13) to be used as radiotracer. Among the [^177^Lu]-(DOTA)-labeled NT(8–13) peptide analogues those containing a short hydrophilic tetraethylene glycol (PEG4) spacer between the amino acid chain and the radiometal complex exhibited the most in vitro promising properties [66]. Moreover, Jia et al. analyzed four different ^177^Lu-Neurotensin analogues with spacer lengths from 1 to 9 atoms (β-Ala-OH (N1), 5-Ava-OH (N2), and 8-Aoc-OH (N3)) between the DOTA and the peptide. All of them showed lower IC_50_ values than the Neurotensin analogue without a spacer (N0). Particularly N1 revealed higher retention and rapid internalization in HT-29 cells and excellent accumulation in the NTR1-positive tumors xenograft in mice by SPECT/CT imaging studies [78].

The results of these studies demonstrate that high flexibility caused by spacers confers higher specificity to radiolabeled moieties for the cognate receptor both in vitro and in vivo.

### 3.2. Radionuclides

Radionuclides for therapy are α-particle emitters or β-particle emitters, such as ^90^Y, ^177^Lu, ^188^Re, ^186^Re, ^67^Cu and ^64^Cu, ^212^Bi,^213^Bi, ^211^At, ^225^Ac and ^131^I [79].

Radiopharmaceutical peptides for clinical diagnosis by SPECT radioimaging are mainly labeled with γ emitters such as ^99m^Tc, ^67^Ga, ^111^In, while useful positron emitters for PET radioimaging includes ^68^Ga, ^64^Cu and ^18^F.

All metallic radionuclides require a chelator for peptide conjugation, while non-metallic radionuclide could be conjugated by direct labeling or via prosthetic groups. The optimal bifunctional chelators should have high thermodynamic stability, kinetic inertness and should produce a minimum number of isomers while reacting with a metal chelate. Moreover, they should have high hydrophylicity to improve blood clearance and renal excretion as well as resistance to radiolysis caused by large doses of β-radiation. A variety of bifunctional chelators have been developed, conjugated to peptides and radiolabeled in order to achieve this goal.

#### 3.2.1. Technetium Chelators

**Figure 2:**
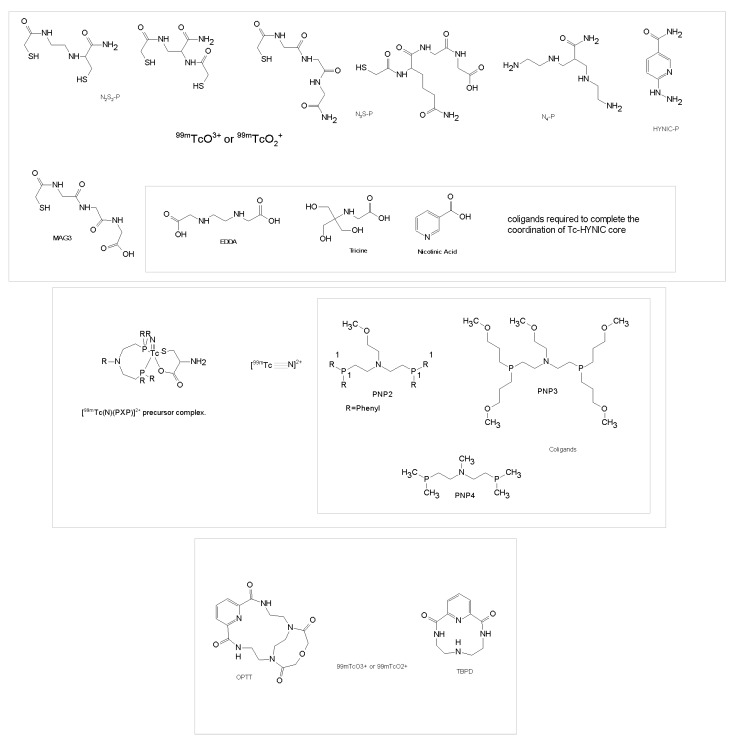
Schematic model illustrating ^99m^Tc chelators and co-ligands.

Labeling of peptides with ^99m^Tc is usually based on ligands containing N_2_S_2_ diamidedithiols, N_3_S triamidethiols, N4 tetraamines [80,81], hydrazine nicotinic acid (HYNIC) [82,83,84] or phosphines [85,86,87,88] (Figure 2). Labeling reactions require high temperatures (80–100 °C), except for N_4_ which reacts at room temperature. ^99m^Tc forms penta- or hexa-coordinated complexes containing TcO^3+^(N_2_S_2_ and N_3_S) or TcO^2+^(N_4_) core. One or more co-ligands containing amines, generally ethylenediaminediacetic acid (EDDA) or *N*-(2-hydroxy-1,1-bis(hydroxymethyl)ethyl)glycine (tricine) and nicotinic acid, are used to label Tc(V)-HYNIC complex. The introduction of HYNIC can be considered as an additional breakthrough in Tc-coordination chemistry since it establishes new parameters causing the formation of stable complexes with the radiometal superior to polydentate ligands. Tridentate chelators such as picolylaminediacetic acid (PADA) or (NαHis)Ac have shown to be superior to bidentate such as the His in the His-peptide conjugate, where one water molecule remains in the coordination sphere [89].

The [Tc≡N]^2+^ core is isoelectronic with [Tc=O]^3+^, stabilizes oxidation state of Tc(V), forms Tc(V)-nitrito complexes with various chelators and has been used to label small peptides [69]. The PXP bisphosphine have been used as coligands to stabilize the core while bifunctional chelators containing thiolate-S, amine-N or carboxylate-O donors are attached to the peptides [90]. It has been demonstrated that the [^99^mTcN(PXP)]^2+^ fragment reacts with the cysteine residue to form asymmetrical ^99m^Tc-nitrido complexes with very high specific activity [88,91,92].

Mercaptoacetyltriglycine (MAG3) (Figure 2) labeled with ^99m^Tc is used for imaging of kidney function, while radiorhenium (^186/188^Re) MAG3 complex has been employed for tumor radiation therapy [78]. Moreover, Wang et al. reported that the bifunctional *N*-hydroxysuccinimidyl ester of MAG3 with *S*-acetyl protection (*N*-hydroxysuccinimidyl *S*-acetylmercaptoacetyltriglycinate (NHS-MAG3)) can covalently conjugate a MAG3 chelator to primary amine functionalized biomolecules without the use of coligands [93].

Tetraazamacrocycle derivatives are widely studied as bifunctional chelating agents for conjugating different biomolecules with radionuclides in target-specific radiotherapy. The novel tetraazamacrobicyclic chelator 3,6,9,15-tetraazabicyclo[9.3.1]pentadeca-1(15),11,13-triene-2,10-dione (TBPD) and pentaazamacrotricyclic chelator 9-oxa-3,6,12,15,21-pentaazatricyclo[15,3,2,1] trieicos-1(21), 17,19-triene-2,7,11,16-tetradione (OPTT) (Figure 2) were synthesized and radiolabeled with ^99m^Tc to produce ^99m^Tc-TBPD and ^99m^Tc-OPTT [94]. These radiolabeled complexes showed high yield, purity, labeling efficiency and in vitro stability. The study of biodistribution showed that the excretory pathway of ^99m^Tc-TBPD was hepatobiliary and of ^99m^Tc-OPTT was renal as well as hepatobiliary. The analysis of TBPD and OPTT cytotoxicity showed no anti-proliferative activity on human cervical SW756, HeLa, and glioblastoma U-87 and U373 cell lines [94]. Overall, these radiolabeled compounds are promising candidates for further development of target-specific imaging agents.

Zhao et al. utilized HYNIC-NHS as chelator for conjugation of isoDGR peptide with ^99m^Tc by using TPPTS as reducing agent together with the coligand tricine to prepare ^99m^Tc-HisoDGR imaging probe for glioma diagnosis [95]. The SPECT/CT imaging experiments on small animals showed a clear visualization of the tumors in subcutaneous and orthotopicglioma tumor models with clear background. The accumulation of ^99m^Tc-HisoDGR in the tumor was significantly reduced by the coinjection of excess cold isoDGR peptide suggesting that the tumor uptake was specific. The ^99m^Tc-HisoDGR could be a promising radiotracer for tumor diagnosis and prognostic evaluation as well as for selecting groups of patients to be enrolled in clinical trials to assess their sensitivity to integrin α5β1-targeted drugs [95].

#### 3.2.2. Yttrium, Indium, Gallium, Lutetium, and Copper Chelators

**Figure 3:**
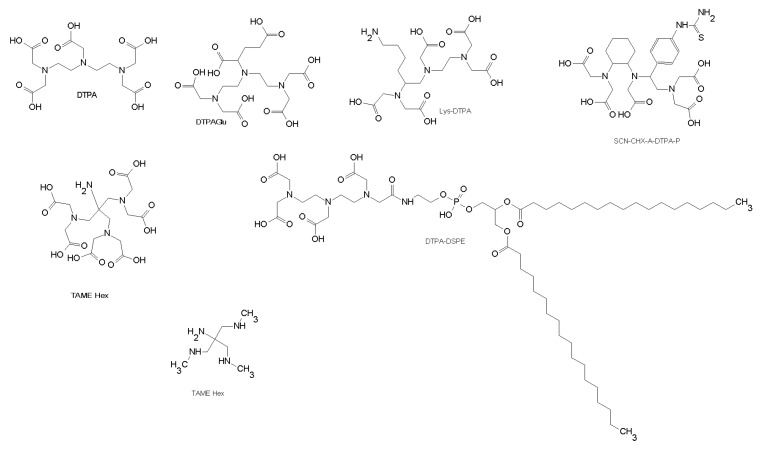
Chemical structures of DTPA chelators.

**Figure 4:**
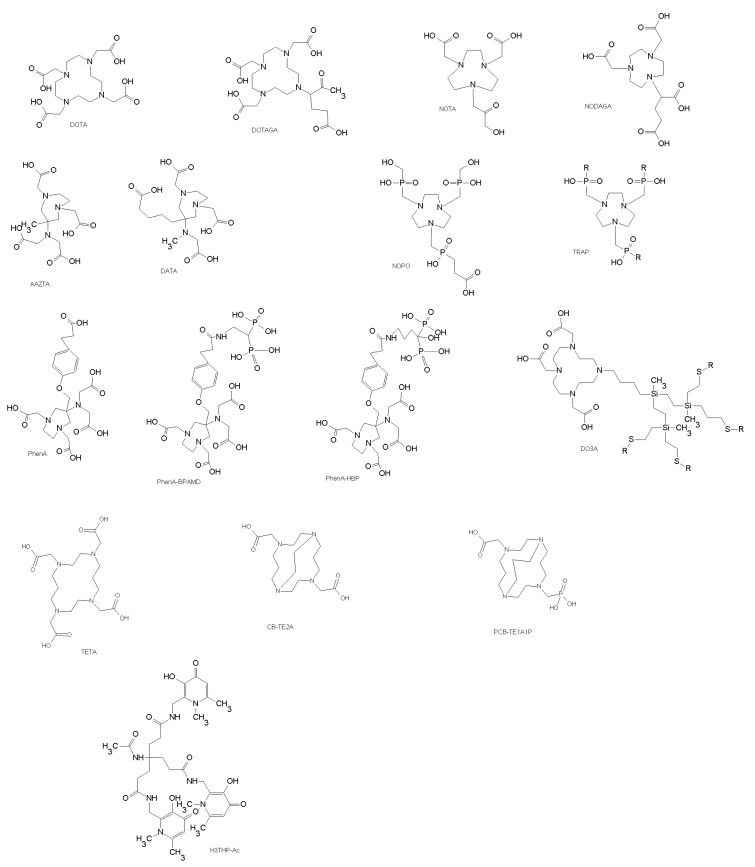
Chemical structures of DOTA chelators and analogues.

The most commonly used chelating agents are polyaminopolycarboxylic ligands with a branched (DTPA like) (Figure 3) or a cyclic (DOTA like) structure (Figure 4). Both DTPA and DOTA are usually coupled to peptides using one carboxylic acid group forming an amine bond. Since DTPA forms stable complexes and has fast labeling kinetic it is still one of the most important bifunctional chelators for ^111^In labeling of peptides. Numerous DTPA derivatives have been developed as chelators for ^68^Ga, ^80^Y, and ^111^In ^177^Lu, ^213^Bi and ^90^Y. For example, DTPAGlu or CHX-A-DTPA (Figure 3), compared to DTPA, have the additional advantage that all five carboxylate groups are preserved for metal binding [96].

The chelator 1,1,1-tris(aminomethyl)ethane (TAME) (Figure 3) has been used as building block that offers nine donor atoms for the complexion of radiometals. The synthesis of bifunctional chelating agent TAME-Hex (Figure 3) has been described and it has shown to have high stability for gallium complexes. In fact, the new gallium chelates are stable against trans-chelation by a 1000-fold excess of DTPA and thus are potentially highly effective candidates for use in radioimaging [97].

To improve therapeutic efficacy of water-soluble drugs DTPA has been incorporated in liposome (DTPA-DSPE) (Figure 3) and radiolabeled with ^111^In-oxine for in vivo tracking. Accardo et al. developed a drug-delivery system, DSPC/*Mon*Y-Bombesin, based on liposomes functionalized with the bioactive sequence of the bombesin analog conjugated with a chelating agent [98]. This drug delivery system could be useful for treatment of patients with tumors expressing gastrin-releasing peptide receptors (GRPRs) at high density, such as ovarian, breast, and prostate cancer [98,99].

Bifunctional chelators containing a macrocyclic core, such as 1,4,7,10-tetraazacyclododecane-1,4,7,10-tetraacetic acid (DOTA) (Figure 4), form metal complexes with high thermodynamic stability and kinetic inertness. However, the radiolabeling kinetics of DOTA-based bifunctional chelators is usually slow, and much more dependent on the radiolabeling conditions, including DOTA-conjugate concentration, pH, reaction temperature and heating time, buffer agent and buffer concentration, and presence of other metal ions, such as Fe^3+^ and Zn^2+^. Several DOTA-peptide and NOTA-peptide conjugates have been labeled with gallium and indium and used in diagnostic imaging like the somatostatin conjugates DOTA-TOC and DOTA-TATE, bombesin analogs, RGD analogs, minigastrin analogs etc.

NOTA and derivatives are well-known to form a very stable complexes with ^68^Ga and with ^64^Cu [100,101]. Coordination of ^68^Ga with DOTA derivatives is less efficient, often requiring heating (80–100°C). Formation of DOTA ligands with ^68^Ga is more sensitive to experimental conditions than that of NOTA analogues (NOTA = 1,4,7-triazacyclononane-1,4,7-triacetic acid) [101]. Derivatives of NOTA, especially NODAGA proved to be more suitable for chelating the ^68^Ga ion than those of DOTA [102].

More recently, a new chelator, the 6-[Bis(carboxymethyl)amino]-1,4-bis(carboxymethyl)-6-methyl-1,4-diazepane (AAZTA) (Figure 4), showed better properties than 1,4,7,10-tetraazacyclododecane-1,4,7,10-tetraacetic acid (DOTA) for radiopharmaceutical applications. The radiolabeling is carried out under mild conditions for short time with trivalent metals including ^68^Ga for PET and ^177^Lu or ^111^In for SPECT and radionuclide therapy [103]. Pfister at al. conjugated the minigastrin sequence peptide with an AAZTA derivative through an aliphatic linker (AAZTA-MG), labeled with ^68^Ga, ^177^Lu and ^111^In and obtained high radiochemical yields at room temperature. The mild reaction conditions preserved the structure of peptides and the biological activity. The radiolabeled AAZTA-MG has shown good tumor targeting although was observed some degradation in human plasma and a considerable uptake by intestine and liver in healthy mice. Additional modifications of chelator structure or linker could improve the tumor targeting and pharmacokinetic properties [103].

Wu et al. obtained an AAZTA derivative, containing a phenol carboxylic acid (PhenA, 2), which is an effective chelator for the radiometal^68^Ga [90]. The ^68^Ga-PhenA bisphosphonates, PhenA-BPAMD, 3, and PhenA-HBP, 4, bind to hydroxyapatite on active bone surfaces while the AAZTA-chelating group forms a stable complex with ^68^Ga. Thus, they may be useful as bone imaging agents for detecting tumor metastases [104].

Seemann et al. synthesized the bifunctional chelator, DATA ligand (2,2′-(6-((carboxymethyl)amino)-1,4-diazepane-1,4-diyl)diacetic acid)) (Figure 4), conjugated to [DPhe1][Tyr3]-octreotide (TOC) and radiolabeled with purified ^68^Ga [105]. The radiotracer showed high stability in the human serum [106]. The DATA chelator rapidly forms stable complexes with ^68^Ga under exceptionally mild conditions befitting kit-type labeling [107].

Triethylenetetramine (TETA) and TETA derivatives have been used for ^64/67^Cu labeling of different peptides. To avoid the in vivo instability of these class of chelators two carboxymethyl pendant arms have been added to cross-bridged (CB)-cyclam obtaining CB-TE2A (Figure 4). CB-TE2A coupled to peptides and labeled with ^64^Cu showed superiority compared to more conventional chelators such as DOTA and TETA [108]. Chelators with cross-bridged cyclam backbones present different advantages, such as high stability of the radiotracer, high efficiency of radiolabeling, and good biological inertness of the radiolabeled complex, along with rapid body clearance. A new generation of propylene-cross-bridged chelators with hybrid acetate/phosphonate pendant groups (PCB-TE1A1P) (Figure 4) has been developed to improve the in vivo stability and fast clearance. PET analysis in mice confirmed that PCB-TE1A1P has good potential as a bifunctional chelator for ^64^Cu-based radiopharmaceuticals, especially those based on peptides [109].

A set of bifunctional chelating agents (BFCAs) based on ionic carbosilane dendrons useful for several biomedical applications was synthesized by Moreno et al. [110]. The presence of the dendritic wedge as a substituent of the DOTA ligand originating DO3A ligands affords the strengthening of the chelate N4 system with respect to the DOTA ligand, while the opposite is observed for the cyclen ligand.

Therefore, the dendron branched (Figure 4) may modify the complexation capacity of the macrocyclic ring compared to that of the DOTA ligand. The presence of such coordinating groups at the branches and periphery of the dendrons may be detrimental for some biomedical purposes such as diagnostic medicine. For istance, loading the dendrons with radioactive metal centers might result in some metal ions being weakly bound to the dendrons.

The weak coordination mode of the peripheral sulfonate or protonable amine groups at the respective dendrons, radioactive metal centers, can be easily located at the macrocycle fragment, leaving the dendritic surface for extra advantage, i.e., increasing solubility, interaction with nucleic acids in the case of amine-terminated dendrons or antiviral properties for anionic sulfonate-terminated dendrons.

To simplify the ^68^Ga labeling procedures novel bifunctional chelators have been designed such as TRAP and NOPO derived from 1,4,7-triazacyclononane macrocycles substituted with phosphinic acid groups at the amine group (Figure 4). Notni et al. showed that TRAP ligands are optimal for the production of ^68^Ga tracers as they allow highly efficient ^68^Ga^3+^complexation in a wide pH range (0.5–5) and require 10–30-fold lower concentrations than NOTA and DOTA for imaging by PET [111]. The TRAP-based cyclo(RGDfK) trimer, ^68^Ga-TRAP-(RGD)3, was shown to have 7-fold higher affinity to αvβ_3_ integrin expressed on M21 human melanoma cells compared to the monomers ^18^F-Galacto-RGD and ^68^Ga-NODAGA-c(RGDyK) [112]. Moreover, the cyclic pentapeptidecyclo(RGDfK) conjugated to NOPO, 1,4,7-triazacyclononane-1,4-bis[methylene (hydroxymethyl)phosphinic acid]-7-[methylene(2-carboxyethyl)phosphinic acid], a bifunctional chelator with optimal ^68^Ga labeling properties , showed high affinity to α_v_β_3_ integrin in vitro and specific uptake in M21 tumor xenografts in vivo [71].

Ma et al. synthesized two bifunctionaltris(hydroxypyridinone) (THP) chelators, each with pendant isothiocyanate groups and three 1,6-dimethyl-3-hydroxypyridin-4-one groups, characterized by rapid labeling with ^68^Ga under mild conditions without the need of subsequent purification (Figure 4) [113]. The effective labeling of THP chelators at very low concentration under mild conditions opens the possibility to label many sensitive proteins with ^68^Ga to produce PET tracers by avoiding complex multistep radiochemistry reactions.

#### 3.2.3. Fluorine and Iodine Prosthetic Groups

**Figure 5:**
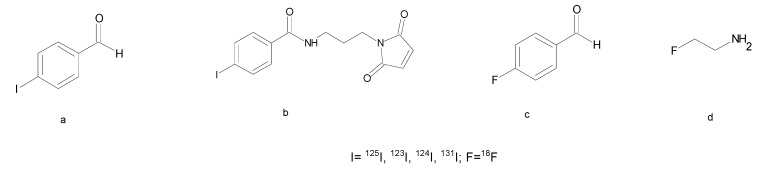
Chemical structures of prosthetic groups for radioiodination and fluorination of peptides: (**a**) 4-[I]iodobenzaldehyde; (**b**) *N*-[2-(2,5-Dioxo-2,5-dihydro-pyrrol-1-yl)-ethyl]-3-[I]iodo-benzamide; (**c**) 4-[F]fluorobenzaldehyde; (**d**) [F]fluoroethylazide.

Fluorination and Iodination are usually performed by nucleophilic substitution into aromatic molecules and electrophilic aromatic substitution on tyrosine or histidine side chains, respectively. However, the fluorination by direct incorporation of [^18^F]fluoride in synthetic peptides is hampered by unfavorable conditions that could affect the stability of peptide structures such as the high temperature, organic solvents or basic conditions. When direct labeling is not possible, the use of prosthetic groups functionalized with reactive molecules such as amine, thiol or aminoxy group is essential to bind [^18^F]fluoride or Iodine to peptides under mild conditions [114]. The aldehydes, such as 4-[Iodo/Fluoro]benzaldeyde, have been successfully used to radioiodinate the multimeric RGD peptides (Figure 5a) [115]. Moreover, the maleimide group, allowing a chemoselective conjugation to thiol peptides, has been used for the radioiododestannylation followed by conjugation to a Cys-peptide under very mild conditions (Figure 5b) [116]. Several other ^18^F-labeling methods have been developed for fast peptide labeling such as chemoselective conjugation methods with aldehydes, alkyne or azide derivatives (Figure 5c). However, the high lipophilicity of the resulting fluorinated radiopeptides generally causes high unspecific liver accumulation and low tumor uptake. Radiolabeled peptides with favorable pharmacokinetics and lower lipophilicity are generally obtained by glycosylation or polyethylene glycol (PEG) conjugation (Figure 5d) [114].

## 4. Conclusions

The discovery of small peptides able to bind specific receptors preferentially expressed on diseased tissues led to the development of synthetic analogues with high affinity to their targets. The recent advances in radiochemistry, the synthesis of new bifunctional chelators and linkers as well as advances in the production of radionuclides resulted in the production of many radiopeptide candidates for tumor targeting.

The peptide cyclization and use of non natural amino acids has been very important to stabilize the peptides in the body fluids and to increase the receptor affinity. The addition of biologically components such as PEG and glycolic molecules has been useful to modify the biodistribution and the bioavailability of the targeting moiety. Indeed since the receptor binding domains are all different there are many possibility to target them with many new synthetic molecules.

## Figures and Tables

**Table 1:** Chemical composition of peptide-based radiopharmaceuticals.

Receptor	Cancer	Peptide	
Somatostatin (sst1/sst2/sst3/sst4/sst5)	Neuroendocrine tumors	***Agonists***	
		Somatostatin	AGCKNFFWKTFTSC (Cys-Cys cyclization)
		Octreotide	(d-F)CF(d-W)KTCT-ol (Cys-Cys cyclization)
		Y3-OC	(d-F)CY(d-W)KTCT-ol (Cys-Cys cyclization)
		TATE	(d-F)CF(d-W)KTCT-OH (Cys-Cys cyclization)
		Y3-TATE	(d-F)CY(d-W)KTCT-OH (Cys-Cys cyclization)
		Lanreotide	(d-2-Nal)CY(d-W)KVCT-NH_2_ (Cys-Cys cyclization)
		Depreotide	cyclo-[(*N*-Me)Phe-Y-d-Trp-KV-Hcy]CH_2_-CO.*β*-Dap-KCK-NH_2_
		Pep2	AGCKNF(l-Dfp)(d-W)KTFTSC [l-Dfp7, d-Trp8,]-SRIF
		Pep3	AGCKNFF(d-Trp)KT(l-Dfp)TSC [d-Trp8, l-Dfp11]-SRIF
		***Antagonists***	
		BASS	p-NO_2_-F((d-C)Y(d-W)KTC)(d-Y)-NH_2_ (Cys-Cys Cyclization)
		LM3	p-Cl-F((d-C)Y(d-Aph(Cbm)KTC)(d-Y)-NH_2_ (Cys-Cys Cyclization)
		JR10	p-NO_2_-F(d-C)Y(d-Aph(Cbm))KTC)(d-Y)-NH_2_ (Cys-Cys Cyclization)
		JR11	p-Cl-F((d-C)-Aph(Hor)-(d-Aph(Cbm))KTC)(d-Y)-NH_2_ (Cys-Cys Cyclization)
VPAC1/VPAC2	Primary and metastatic breast, ovary, prostate, colon, bladder carcinomas, meningiomas	VIP	HSDAVFTDNYTRLRKQMAVKKYLNSILN
			HSDAVFTRNYTRLRRQLAVKRYLNSILN-NH_2_
			VIP, [R8,15,21, L17]-VIP
CCK1/CCK2	Gastrointestinal stromal tumor, stromal ovarian cancer, astrocytomas, medullary thyroid carcinomas	CCK analogs	DYMGWMDF-NH_2_
			DOTA-K(glucose)-GW-Nle-DF
			DOTA-Nle-cyclo(EW-Nle-DFK)-NH_2_
		Minigastrin	(d-E)AYGWMDF-NH_2_
			l-(E)5-AYGWMDF-NH_2_
			(d-E)E(5)AYGWMDF-NH_2_
BB1, BB2, BB3, BB4	Prostate and breast cancer, glioma	Bombesin	pGlu-QRLNQWAVGHLM-NH_2_
			pGlu-QRLNQWAVGH-Cha-NLeu-NH_2_
			pGlu-QRLNQWAVG-Cha-Sta-Nleu-NH_2_
			pGlu-QRLNQWAV-Sta-NMeGly-Nleu-NH_2_
			pGlu-QRLNQWAVGH(d-Phe)M-NH_2_
hMC-1R, hMC-3R, hMC-5R	Melanogenesis	α-MSH	Ac-SYSMEHFRWGKPV
			Ac-GGNle-CCEH(d-F)RWC-NH_2_
			Ac-GGNle-CCEH(d-F)RWCRP-NH_2_
			Ac-CCEH(d-F)RWC-NleGG-NH_2_
			Ac-CCEH(d-F)RWCRP-NleGG-NH_2_
NTR_1_, NTR_2_, NTR_3_	Tumor progression (lung cancer, breast cancer, prostate cancer)	Neurotensin	ELYENKPRRPYIL
			H-KKPYI-TMS-A-OH
			RRPYIL
			PEG4-RRPYIL
			PEG4-RRPYIL
			PEG4-RKPY-Tle-L
			PEG4-KRPY-Tle-L
			PEG4-KKPY-Tle-L
Integrins	angiogenesis	RGD analogs	RGD
			RGDS
			GRGDS
			GRGDPS
			GRGDSPK
			c(RGDxX) x = d-Phe, d-Tyr, d-Trp; X = K, C, V
			c(FRGDLAFp(NMe)K)
			FR366 *
			c(RGDfK) trimer
GRP78	Cervix, esophagous, pancreas, lung and glioma tumors	GRP78	GIRLRG
			PEG-GIRLRG

* 2,2′-(7-(1-carboxy-4-((6-((3-(4-(((*S*)-1-carboxy-2-(2-(3-guanidinobenzamido)acetamido)ethyl)carbamoyl)-3,5-dimethylphenoxy)propyl)amino)-6-oxohexyl)amino)-4-oxobutyl)-1,4,7-triazonane-1,4-diyl)diacetic acid.
